# Preserved endothelium-dependent dilatation of the coronary microvasculature at the early phase of diabetes mellitus despite the increased oxidative stress and depressed cardiac mechanical function *ex vivo*

**DOI:** 10.1186/1475-2840-12-49

**Published:** 2013-03-25

**Authors:** Evangelia Mourmoura, Guillaume Vial, Brigitte Laillet, Jean-Paul Rigaudière, Isabelle Hininger-Favier, Hervé Dubouchaud, Beatrice Morio, Luc Demaison

**Affiliations:** 1 Laboratoire de Bioénergétique Fondamentale et Appliquée, INSERM U1055, Université Joseph Fourier, BP 53, Grenoble cedex 09 F-38041, France; 2 Université Joseph Fourier, Laboratoire de Bioénergétique Fondamentale et Appliquée, INSERM U1055, Grenoble cedex 09 F-38041, France; 3 INRA, Clermont Université, Université d’Auvergne, Unité de Nutrition Humaine, BP 10448, Clermont-Ferrand, F-63000, France

**Keywords:** Diabetes mellitus, Insulin resistance, Coronary reactivity, Microvasculature, Mechanical function, Oxidative stress

## Abstract

**Background:**

There has been accumulating evidence associating diabetes mellitus and cardiovascular dysfunctions. However, most of the studies are focused on the late stages of diabetes and on the function of large arteries. This study aimed at characterizing the effects of the early phase of diabetes mellitus on the cardiac and vascular function with focus on the intact coronary microvasculature and the oxidative stress involved.

**Materials and methods:**

Zucker diabetic fatty rats and their lean littermates fed with standard diet A04 (Safe) were studied at the 11th week of age. Biochemical parameters such as glucose, insulin and triglycerides levels as well as their oxidative stress status were measured. Their hearts were perfused *ex vivo* according to Langendorff and their cardiac activity and coronary microvascular reactivity were evaluated.

**Results:**

Zucker fatty rats already exhibited a diabetic state at this age as demonstrated by the elevated levels of plasma glucose, insulin, glycated hemoglobin and triglycerides. The *ex vivo* perfusion of their hearts revealed a decreased cardiac mechanical function and coronary flow. This was accompanied by an increase in the overall oxidative stress of the organs. However, estimation of the active form of endothelial nitric oxide synthase and coronary reactivity indicated a preserved function of the coronary microvessels at this phase of the disease. Diabetes affected also the cardiac membrane phospholipid fatty acid composition by increasing the arachidonic acid and n-3 polyunsaturated fatty acids levels.

**Conclusions:**

The presence of diabetes, even at its beginning, significantly increased the overall oxidative stress of the organs resulting to decreased cardiac mechanical activity *ex vivo*. However, adaptations were adopted at this early phase of the disease regarding the preserved coronary microvascular reactivity and the associated cardiac phospholipid composition in order to provide a certain protection to the heart.

## Background

The prevalence of Type 2 Diabetes (T2D) is increasing at an alarming rate assuming epidemic dimensions in industrialized societies [1]. Individuals with T2D have increased risk for developing cardiovascular diseases (CVDs), which is the main cause of early mortality and morbidity in the Western world [2]. Insulin resistance and T2D usually result from excess intake of deleterious nutrients such as saturated and trans fatty acids [3]. Consequent metabolic changes such as hyperinsulinemia and hyperglycemia [4] can provoke vascular lesions and endothelial dysfunctions at both micro- and macro-circulations [5-8]. The vulnerability of the coronary circulation to the diabetic milieu can lead to endothelial dysfunction at this bed, which consists a significant biomarker of early coronary artery disease independently of atherosclerosis [9].

An accurate experimental model in order to clarify the mechanisms responsible for the pathophysiology of diabetes evolution and its complications is the inbred Zucker diabetic fatty (ZDF) rat. The homozygous fa/fa Zucker rat exhibits hyperphagia caused by a non-functioning leptin receptor. This leads to the development of obesity, hyperglycemia, hyperinsulinemia and finally diabetes at a young age [10,11]. Previous studies on these rats in the later stages of diabetes have demonstrated that chronic hyperglycemia and hyperlipidemia can result in inflammation [12,13], increased oxidative stress and vascular dysfunction [14,15]. Coppey *et al.*[16] have shown that the endothelium-mediated responses to acetylcholine (Ach) are attenuated in epineurial arterioles of the sciatic nerve in diabetic ZDF rats. A key feature is the reduced production of nitric oxide (NO), a compound which mediates endothelium-dependent vasorelaxation and inhibits inflammation. In T2D, its bioavailability can be diminished either by the impaired insulin signaling either by the action of reactive oxygen species (ROS) [17].

Although the consequences of the later stages of T2D on the cardiac and endothelial function are well characterized, less is known concerning the early period of the disease where an interventional treatment may be more effective. Furthermore, most studies are focused on the endothelial function and perfusion of large arteries [18-20] and few on the coronary function of resistance vessels. The primary function of the coronary microcirculation is to optimize nutrient and oxygen supply to the heart in response to any metabolic demand by coordinating the resistances within different microvascular domains, each governed by distinct regulatory mechanisms [21]. Coronary resistance arteries are capable of adapting to acute or chronic increases in blood flow leading to an increased NO-mediated relaxation and a consequent enlargement of their diameter. Furthermore, endothelial dysfunction in resistance arteries seems to precede that of large arteries [22]. Although there is strong evidence indicating that T2D is associated with impaired vasodilator responses of both peripheral and coronary vessels, Oltman *et al.*[22] have demonstrated that in diabetic young (8- to 12 wk old) ZDF rats the coronary arteriolar dilation to Ach of isolated microvessels is preserved. However, these *in vitro* studies isolate the coronary system from the cardiac environment and its influences.

Thus, this work aimed at studying the *ex vivo* cardiac and coronary vascular functions of young ZDF rats and at characterizing the levels of oxidative stress in their organs. The endothelial function of the intact coronary microvasculature was assessed in terms of endothelium-dependent and -independent vasodilatations in an *ex vivo* heart perfusion model at this phase of T2D. The NO production in aortas and hearts was evaluated indirectly by estimating the degree of phosphorylation of the endothelial NO-synthase (eNOS) at serine 1177 (Ser1177) and the iNOS levels in the heart. Finally, the fatty acid profile of cardiac membrane phospholipids was evaluated since any modification at this level leads to functional changes in lipid-protein interactions and related signaling pathways.

## Methods

### Animals and experimental design

All experiments followed the European Union recommendations concerning the care and use of laboratory animals for experimental and scientific purposes. All animal work was approved by the local board of ethics for animal experimentation (Cometh) and notified to the research animal facility of our laboratory (authorization n° 38 07 23). The performed research was in compliance with the ARRIVE guidelines on animal research [23].

Ten ZDF and eleven Zucker lean (ZL) male rats were obtained from Charles Rivers (L’Arbresle, France) at 7 weeks (wk) of age. Rats were housed in a temperature- and humidity-controlled facility on a 12-h light:dark cycle. The two groups were fed *ad libitum* with a standard carbohydrate diet (A04, Safe, Augy, France), they had free access to water and their body weight and food intake were recorded twice weekly. The composition of the chosen diet by weight is 60% assimilable glucides (52% mainly starch and cellulose), 16% proteins and 3% fat. After analysis of the fatty acid composition of our diet we found a formula with approximately 24% of saturated fatty acids (SFAs), 23% of monounsaturated fatty acids (MUFAs), 48% of n-6 polyunsaturated fatty acids (PUFAs) and 4.5% of n-3 PUFAs. Plasma glucose and glycated hemoglobin (HbA1c) concentrations were also measured weekly via the tail vein.

On the day of the experiment, the rats were weighed and heparinized (1500 I.U./kg) intraperitoneally 30 minutes (min) before their decapitation. Blood samples were collected for further biochemical analysis and their retroperitoneal and mesenteric adipose tissues were weighed for determination of the abdominal fat mass [24]. Perfusion experiments were performed twice a day by alternating the rat phenotypes. The first experiment was performed between 8:00 a.m. and 8:30 a.m. and the second one between 12:30 p.m. and 1:00 p.m.

### Cardiac function study

All rats underwent *ex vivo* Langendorff assessment of their cardiac function. For this reason, a rapid thoracotomy was performed and the heart was immediately collected in Krebs-Henseleit solution maintained at 4°C. It was then rapidly (less than one minute to avoid problems of cellular damages or preconditioning) perfused at constant pressure according to the Langendorff mode with a Krebs–Henseleit buffer containing (in mM) NaCl 119, MgSO_4_ 1.2, KCl 4.8, NaHCO_3_ 25, KH_2_PO_4_ 1.2, CaCl_2_ 1.2 and glucose 11 mM as sole energy substrate. The perfusion buffer used was similar for both groups in order to minimize the variables studied. The buffer was maintained at 37°C and continuously oxygenated with carbogen (95% O_2_/5% CO_2_). A latex balloon connected to a pressure probe was inserted into the left ventricle and filled until the diastolic pressure reached a value of 7–8 mmHg. This allowed the monitoring of heart rate, systolic, diastolic and left ventricle developed pressures throughout the perfusion protocol. A pressure gauge inserted into the perfusion circuit just upstream the aortic cannula allowed the evaluation of the coronary pressure. The heart was perfused at constant pressure of 59 mmHg [25] for 30 minutes and the coronary flow for each heart was evaluated by weight determination of 1-min collected samples at the 25th min of perfusion. After this period, the heart was perfused at constant flow conditions, for which the flow rate was adjusted in order to obtain the same coronary flow as in the preparation at constant pressure. The systolic, diastolic and left ventricle developed pressures as well as the heart rate was determined after 10 min of perfusion at forced flow in order to allow a satisfying stabilization of the heart. The left ventricle developed pressure was calculated by subtracting the diastolic pressure to the systolic pressure. The rate-pressure product (RPP) was defined as the product of left ventricle developed pressure and heart rate and was used as indicator of the cardiac mechanical work [26]. All the parameters were recorded and analyzed with a computer using the HSE IsoHeart software (Hugo Sachs Elektronik, March-Hugstetten, Germany).

### Coronary reactivity

After the evaluation of the cardiac function at constant flow, we assessed the effects of diabetes on the coronary reactivity. After the 10-min equilibration period at constant flow, the coronary tone was raised by using the thromboxane analog U46619 (30nM), which was constantly infused into the perfusion system near the aortic cannula at a rate never exceeding 1.5% of the coronary flow. This allowed the obtainment of a coronary pressure between 120 and 130 mmHg. In our model of perfusion at forced flow, the aortic pressure equaled the coronary pressure and changes in the coronary tone triggered modifications of the aortic pressure. Changes in aortic perfusion pressure were thus used to monitor changes in coronary tone. Furthermore, this experimental model allows the evaluation of the coronary microvasculature reactivity since the coronary resistance vessels determine the overall coronary pressure. Relaxation responses to Ach (4, 10, 20, 40, 60, 80 and 100 pmoles) and sodium nitroprusside (SNP, 100, 200, 400, 600, 800 and 1000 pmoles) injections were determined reflecting the endothelial-dependent vasodilatation (EDD) and endothelium-independent vasodilatation (EID) respectively.

The dilatation amplitude was calculated as the ratio between the maximal decrease in the coronary pressure and the coronary pressure just before the injection of the dilatation agents. Since the heart weight and coronary volume were subjected to intra- and inter-group variations, a correction was performed to normalise the input-function of the vasodilatation agents according to the coronary flow. The dose–response curve between the amount of vasodilatation agent injected and the maximal vasodilatation was then fitted to a logarithm function for each heart which allowed the fulfillment of statistical analyses. Moreover, the vasodilatation activity of the endothelial cells was also estimated from the corrected EDD and EID curves. For each heart and each injected Ach dose, the amount of SNP (reflecting the amount of vasodilator agents) necessary to obtain the same percentage of Ach-induced vasodilatation was extracted from the EID curve according to the formula: endothelial cell vasodilatation activity (ECVA) = e ^[(% Ach-induced dilatation - b)/a]^, where a and b are the coefficients of the theoretical EID curve. The results were expressed in pmole equivalents of nitroprusside. At the end of the perfusion protocol, the hearts were freeze-clamped and stored at -80°C until the biochemical analyses were performed.

### Oxidative stress measurements

#### Plasma oxidative stress

Protein oxidation in the plasma was evaluated by the disappearance of protein thiol groups [27]. Plasma thiols were assayed in 20 μl of plasma, using 5,5'-dithiobis(2-nitrobenzoic acid (DTNB)) for deriving the thiol groups. The calibration curve was obtained by mixing two stock solutions of N-acetyl cystein (NAC) in the range of 0.125–0.6 mmol/l. Standards and plasma samples were measured spectrophotometrically at 415 nm (Hitachi 912, B Braun Science Tec, France) in the presence of a phosphate buffer 50 mM, EDTA 100 mM, pH 8 and bis-5,5'-dithio-bis(2-nitrobenzoic acid) 10 mM.

The antioxidant status of the plasma was evaluated using ferric reducing antioxidant power (FRAP) assay as a global marker of the antioxidant power. The FRAP assay uses antioxidants as reductants in a redox-linked colorimetric method. In this assay, at low pH, a ferric-tripyridyltriazine (Fe^III^-TPTZ) complex is reduced to the ferrous form, which is blue and monitored by measuring the change in absorption at 593 nm. The change in absorbance is directly proportional to the reducing power of the electron-donating antioxidants present in plasma. The absorbance change is translated into a FRAP value (in μmol/l) by relating the change of absorbance at 593 nm of test sample to that of a standard solution of known FRAP value.

Glutathione peroxidase (GPx) activity, which is a seleno-enzyme involved in protection against hydrogen peroxide (H_2_O_2_) was evaluated in plasma samples by the modified method of Gunzler [28] using terbutyl hydroperoxide (Sigma Chemical Co, Via Coger, Paris, France) as a substrate instead of hydrogen peroxide. The principle of the assay is based on the coupled reaction with glutathione reductase (GR). Oxidized glutathione (GSSG), produced upon reduction of an organic hydroperoxide by GPx with glutathione (GSH) as electron donor, is recycled to its reduced state by GR and NADPH. The oxidation of NADPH to NADP^+^ is accompanied by a decrease in absorbance at 340 nm. The rate of decrease in the A340 is directly proportional to the GPx activity in the sample. The assay was performed at 25°C and pH 7.0 that allowed a stable concentration of GSH in the reaction medium.

### Cardiac oxidative stress

Lactate and pyruvate released in the coronary efflu-ents were spectrophotometrically assayed according to Bergmeyer [29]. The lactate to pyruvate ratio was calculated to estimate the cytosolic redox potential (NADH/NAD^+^) [30-32]. This is a highly specific assay using the enzyme lactate dehydrogenase (LDH) to catalyze the reversible reaction of pyruvate and NADH to lactate and NAD^+^. The catalytic action of LDH permits spectrophotometric measurement at 340 nm (spectrophotometer ULTROSPEC^TM^ 2100 pro, Amersham Biosciences, Uppsala, Sweden) of lactate production in terms of the generation of NADH in the reaction shown above. To measure lactate, the reaction is carried out from right to left with excess NAD^+^. To force the reaction to completion in this direction, it is necessary to trap formed pyruvate with hydrazine. The increased absorbance at 340 nm due to NADH formation becomes a mole-to-mole measure of the lactate originally present in the sample.

Lipid peroxidation was assessed by measuring the concentration of thiobarbituric acid reactive substances (TBARS) in cardiac homogenates [33]. TBARS were determined using the fluorimetric determination of malondialdehyde – thiobarbituric acid complex after acid hydrolysis at 95°C and extraction with n-butanol. Briefly, tissue homogenate aliquots were placed in polyethylene tubes mixed with TBA (thiobarbituric acid)/perchloric acid 7% (2:1, v/v) and incubated at 95°C. After cooling, n-butanol was added for the extraction and then the aliquots were centrifuged for 10 min at 35000 g. The supernatant was used to read the fluorescence at excitation and emission wavelengths of 532 and 553 nm respectively. The TBARS calculated were normalized to the content of polyunsaturated fatty acids of cardiac membrane phospholipids since it differed between groups. The protein thiol groups and FRAP assays were also evaluated in cardiac homogenates as described previously for the plasma.

### Cardiac mitochondrial oxidative stress

The ratio between the activities of aconitase and fumarase of the myocardium was calculated as an indicator of mitochondrial ROS production. Mitochondrial aconitase is sensitive to inactivation by superoxide due to the susceptibility of its iron-sulfur core to oxidation; however, fumarase is unaffected. Thus, the activity ratio of aconitase to fumarase was calculated as an indicator of the presence of mitochondrial ROS [34]. Aconitase and fumarase activities were determined according to Gardner *et al.*[34], but were measured after extraction with a medium supplemented with citrate sodium (1 M) in order to stabilize the aconitase activity *ex vivo*. Values of aconitase and fumarase activities were determined on the same extract for each biological sample.

### Respiratory chain complexes and citrate synthase activities

Activities of the NADH-ubiquinone oxido-reductase (complex I), succinate-ubiquinone oxido-reductase (complex II), ubiquinol cytochrome c reductase (complex III), cytochrome c oxidase (complex IV), NADH cytochrome c reductase (activity of complex I + III) and succinate cytochrome c reductase (activity of complex II + III) were determined as previously described [35]. Heart samples (100 mg) were homogenized at 4°C with 0.9 ml of a potassium phosphate buffer 100 mM, pH 7.4. The homogenates were centrifuged (1,500 × g, 5 min, 4°C), and the resulting supernatants were stored at -80°C until the determination of the various enzymatic activities. Activity of the citrate synthase was determined according to Faloona and Srere [36]. The activities of the respiratory chain complexes and citrate synthase were expressed in units per mg of proteins.

### Western blot

The expressions of total eNOS, phosphorylated eNOS at Ser1177 and iNOS were evaluated by Western blot. Frozen samples were homogenized in ice-cold lysis buffer containing 20 mM Tris (pH 7.8), 137 mM NaCl, 2.7 mM KCl, 1 mM MgCl2, 1% Triton X-100, 10% (w/v) glycerol, 10 mM NaF, 1 mM ethylenediaminetetraacetic acid, 5 mM Na pyrophosphate, 0.5 mM Na_3_VO_4_, 1 μg/ml leupeptin, 0.2 mM phenylmethylsulfonyl fluoride and 1 mM benzamidine. The homogenates were centrifuged at 5,000 *g* for 20 min at 4°C, and the protein concentration in the supernatant was determined in each aliquot. Protein extracts (50 μg/lane) were loaded onto a 10% SDS gel and separated by electrophoresis. Extracts from the control group were loaded on both gels, and the amount of protein was accordingly compared pairwise. Proteins were transferred to nitrocellulose membranes. The membranes were incubated overnight at 4°C with rabbit antibodies against total eNOS (1:150, Thermoscientific, Illkirch, France), iNOS (1:1,000, BD Biosciences Pharmingen, Le Pont de Claix, France) and phosphospecific mouse antibodies against eNOS Ser1177 (1:1,000, BD Biosciences Pharmingen, Le Pont de Claix, France). After being washed in tris buffered saline (TBS)-Tween, the membranes were incubated with horseradish peroxidase-conjugated anti-mouse IgG for eNOS Ser1177 (1:3000, Jackson Immunoresearch, Montluçon, France) and anti-rabbit IgG for total eNOS and iNOS (1:20000, Jackson Immunoresearch, Montluçon, France) for 1 h at room temperature, followed by additional washing. Proteins were visualized by enhanced chemiluminescence with ECL advanced Western blotting detection kit (Amersham Biosciences, Brumath, France) and quantified using densitometry and Image J software. PAN-Actin (1:1000, Cell Signaling Technology*,* St*-*Quentin-en-Yvelines, France) was used as a loading control.

### RNA isolation

Total RNA from rat hearts was isolated by using Tri reagent according to instruction recommended by the manufacturer. Briefly, 50 mg of tissue was homogenized in 1 ml of Tri reagent (Sigma Chemical Co, Via Coger, Paris, France) and the aqueous phase was collected after chloroform addition. RNA was precipitated with isopropanol and washed with 75% ethanol. The RNA pellet was dried and redissolved in diethylpyrocarbonate water. The concentration and purity of RNA were determined by measurement of absorbance at 260:280 nm. RNA samples (2.5 μg of total RNA) were analyzed by 1% agarose electrophoresis for control of RNA degradation.

### Quantitative real-time PCR

1 μg of total RNA of each sample was primed with oligo(dT) and reverse transcribed with SuperscriptIII reverse transcriptase (RT) (Life Technologies SAS, Saint Aubin, France) to produce cDNA. PCR amplification of the cDNA from the reverse transcription reaction was carried out using specific primer pairs for *Nos2* (nitric oxide synthase 2, inducible; GenBank accession number: NM_012611) and the house-keeping gene *Arbp* (acidic ribosomal phosphoprotein; GenBank accession number: NM_022402.2). Sequences of the primers for analysis of mRNA were for *Nos2* (forward): CAG GTT GAG GAT TAC TTC TTC CA; *Nos2* (reverse): TGT CAG AGT CTT GTG CCT TTG with PCR product length of 132 bp and for *Arbp* (forward): CCT GCA CAC TCG CTT CCT A; *Arbp* (reverse): TGA TGG AGT GAG GCA CTG AG with PCR product length of 95 bp (Eurogentec France SASU, Angers, France). These primers were intron-spanning in order to avoid genomic DNA contamination. Quantitative real-time PCR (qRT-PCR) was then performed with a LightCycler FastStart DNA Master SYBR Green I kit (Roche, Diagnostics, Meylan, France) on a LightCycler 1.5 Instrument (Roche, Diagnostics, Meylan, France) in capillaries of 20 μl volume/capillary by adding 4 μl of Master Mix solution, 0.5 μM of *Nos2* or 0.4 μM *Arbp* primers, 5 μl of the sample and completing up to 20 μl with RNAse free water. The assays were performed in duplicates. The capillaries were then appropriately sealed, centrifuged for few seconds at high speed and then placed into the LightCycler. The thermal cycle conditions were 95°C for 10 min (pre-incubation) followed by 45 cycles of amplification that were run at 95°C for 10 s, at 55°C for 10 s and at 72°C for 10 s for *Nos2* and at 95°C for 10 s, at 55°C for 5 s and at 72°C for 6 s for *Arbp*. Cycle threshold values (Ct) were analyzed and the level of expression of *Nos2* gene was standardized against that of *Arbp* gene detected in the same sample by using the 2^-ΔΔCt^ method [37].

### Fatty acid composition of cardiac phospholipids

The phospholipid fatty acid composition was determined in cardiac homogenates as previously described [38]. The lipids were extracted according to Folch *et al.*[39]. The phospholipids were separated from non-phosphorus lipids using a Sep-pack cartridge [40]. After transmethylation, the fatty acid methyl esters were separated and analyzed by gas chromatography.

### Other biochemical determinations

Blood glucose concentrations were determined with a glucose analyzer (ACCU-CHECK Active, Softclix). Plasma insulin concentrations were determined using a radioimmunoassay kit (ICN Pharmaceuticals, Orangeburg, SC). Plasma triglyceride and cholesterol levels were measured using commercially available kits from Biomérieux (Craponne, France) and Roche (Boulogne-Billancourt, France), respectively. HbA1c levels were evaluated with the kit Bayer Healthcare’s analyser A1cNow® determined in blood samples (5 μl) drawn from the rat fingers. Proteins were measured using the bicinchoninic acid method with a commercially available kit (Thermo Scientific, Rockford, IL).

### Statistical analysis

Results are presented as mean ± S.E.M. Animal weight, heart dry weight, glycemia, activity of respiratory chain complexes, aconitase-to-fumarase and lactate-to-pyruvate ratios and data describing the oxidative stress and the cardiac mechanical and vascular function (developed pressure, heart rate, rate pressure product, coronary pressure, and coronary flow) were contrasted across the two groups by one-way analysis of variance (ANOVA). Measures related to the action of the vasodilatation agents were treated with repeated-measures ANOVA to test the effect of the diabetes of ZDF rats (external factor), that of the amount of dilatation agent (internal factor) and their interaction. When required, group means were contrasted with a Fisher’s LSD test. A probability (p) less than 0.05 was considered significant. Statistical analysis was performed using the NCSS 2004 software.

## Results

### General data

**Figure 1:**
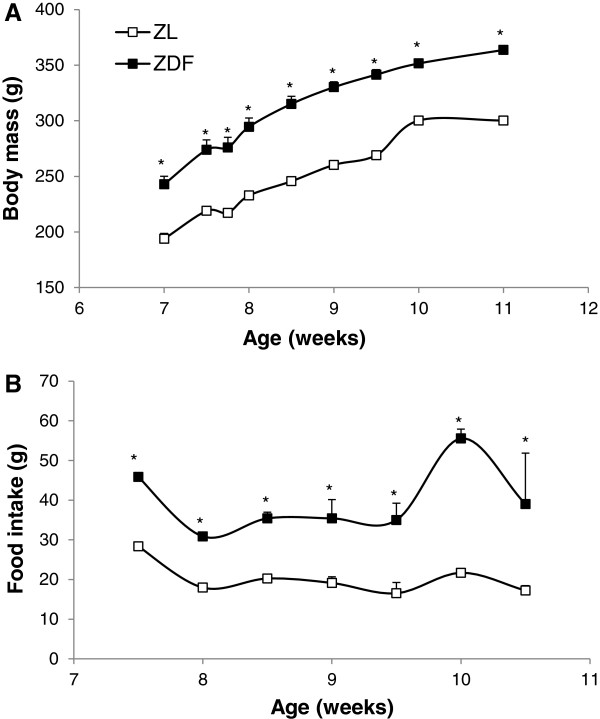
**Evolution of the body weight and food intake of the animals. **(**A**) Body weight and (**B**) Food intake of the animals between the 7th and 11th week of life. ZL: Zucker lean rats; ZDF: Zucker Diabetic Fatty rats. The number of experiments was 11 and 10 for the ZL and ZDF groups respectively. *: significantly different.

As shown in Figure 1, the food intake was always higher in the ZDF compared to the ZL group (+85% at the 9th wk of age), which provoked their increased body weight (+21% at the day of the sacrifice). Consequently, based on the analysis of the diet ingredients, the ZDF group consumed greater amounts of fat than the lean group. The abdominal fat mass was partly responsible for the increased body weight since the mesenteric, retroperitoneal and visceral adipose tissues were significantly heavier as shown in Table 1. However, the heart weight of the ZDF rats did not differ of that of the ZL animals (Table 1).

**Table 1:** Adipose tissue and heart weights

	**ZL**	**ZDF**
Mesenteric AT	1.77 ± 0.09	4.94 ± 0.22*
Retroperitoneal AT	1.04 ± 0.07	4.56 ± 0.13*
Visceral AT	2.81 ± 0.02	9.50 ± 0.32*
Abdominal AT	3.85 ± 0.22	14.06 ± 0.43*
Abdominal AT/BW	0.013 ± 0.001	0.039 ± 0.001*
Heart	203 ± 12	200 ± 6
Heart weight/BW (mg/g)	0.68 ± 0.03	0.55 ± 0.02*

The number of experiments was 11 and 10 for the ZL and ZDF groups respectively. The weight of mesenteric, retroperitoneal, visceral and abdominal adipose tissues is expressed in g of wet weight. The abdominal adipose tissue weight normalized to the body weight is expressed in g of wet weight per g of body weight. The heart weight is expressed in mg of dry weight. The heart-to-body weight ratio is expressed in mg of dry weight per g of body weight. ZL: Zucker lean rats; ZDF: Zucker diabetic fatty rats; AT: adipose tissue; BW: body weight; *: significantly different.

**Figure 2:**
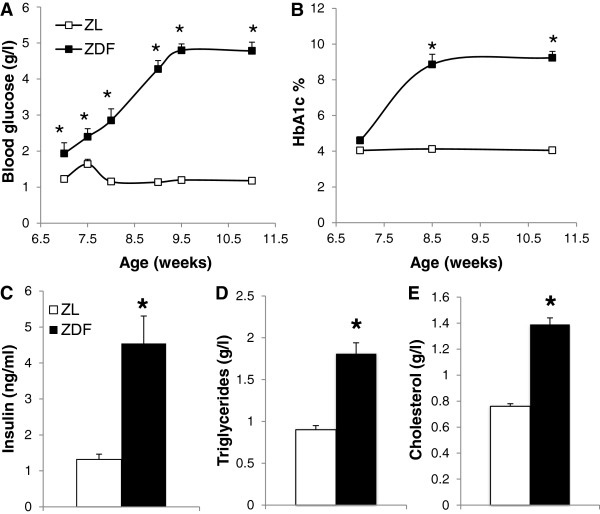
**Evaluation of circulating biochemical parameters. **(**A**) Evolution of blood glucose concentration and (**B**) proportion of glycated hemoglobin (HbA1c) between 7th and 11th wk of life. (**C**) Plasma levels of insulin, (**D**) triglycerides and (**E**) cholesterol at 11th wk of age. ZL: Zucker lean rats; ZDF: Zucker Diabetic Fatty rats. The number of experiments was 11 and 10 for the ZL and ZDF groups respectively. *: significantly different.

Figure 2 shows that the blood glucose concentration of the ZDF rats was increased as soon as the 7th wk of age and reached a value close to 5 g/l at the 9th wk (+276% compared to the ZL rats). The hyperglycemia triggered an increase in HbA1c already significant at the 9th wk (+56%) and a huge augmentation of the insulinemia (+244% at the moment of the sacrifice). The plasma triglyceride and cholesterol concentrations were also significantly higher for the ZDF rats at the moment of the sacrifice.

### Oxidative stress

**Figure 3:**
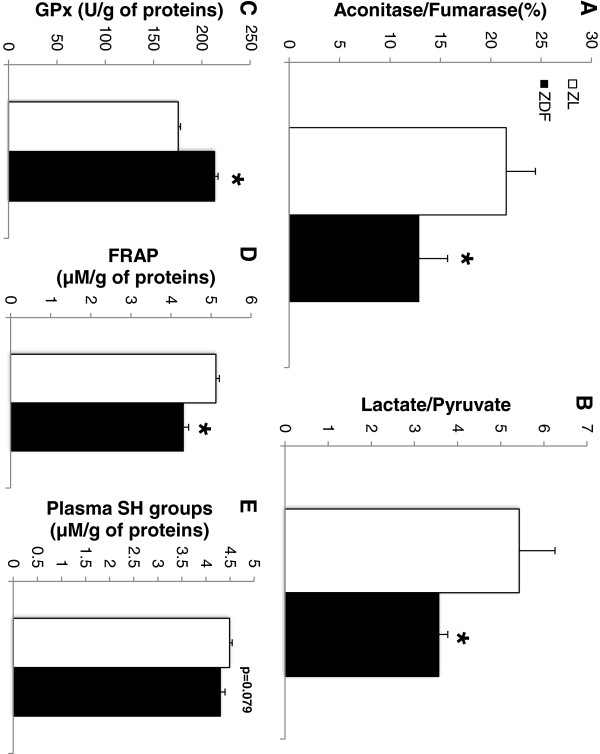
**Oxidative stress measurements. **(**A**) Mitochondrial oxidative stress estimated by aconitase-to-fumarase ratio. (**B**) Cytosolic oxidative stress estimated by lactate-to-pyruvate ratio. (**C**) Enzymatic activity of glutathione peroxidase (GPx) in the plasma. (**D**) Antioxidant power of the plasma estimated by the ferric reducing antioxidant power (FRAP) assay. (**E**) Systemic oxidative stress estimated by the disappearance of the plasma thiol (SH) groups. ZL: Zucker lean rats; ZDF: Zucker Diabetic Fatty rats. The number of experiments was 11 and 10 for the ZL and ZDF groups respectively. *: significantly different.

**Figure 4:**
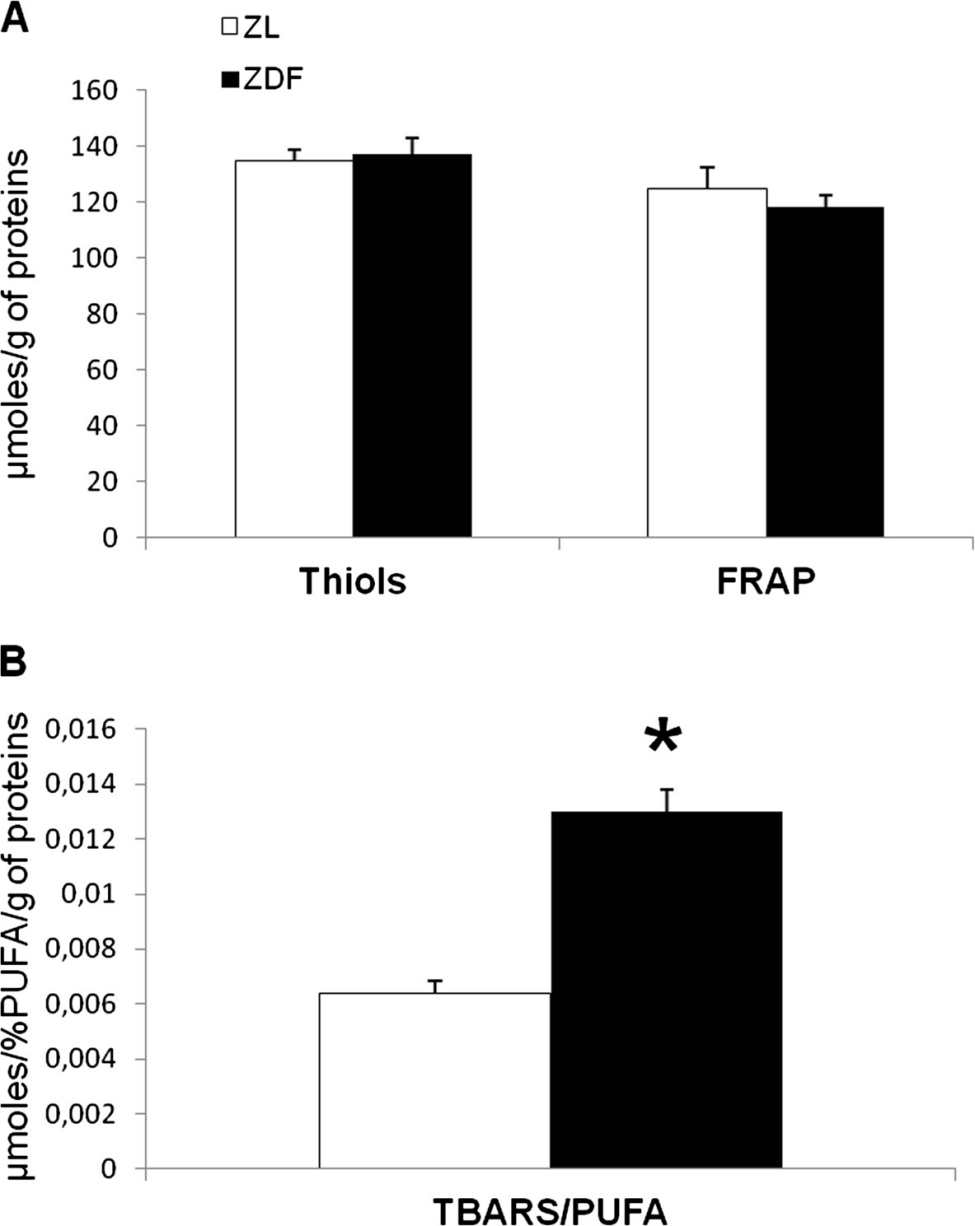
**Oxidative stress in cardiac tissue. **(**A**) Cardiac oxidative stress estimated by the disappearance of the protein thiol (SH) groups and the antioxidant power in cardiac homogenates by the ferric reducing antioxidant power (FRAP) assay. (**B**) Cardiac lipid peroxidation estimated by the thiobarbituric reducing substances (TBARS) assay normalized to the polyunsaturated fatty acid content of cardiac membrane phospholipids. ZL: Zucker lean rats; ZDF: Zucker Diabetic Fatty rats. The number of experiments was 11 and 10 for the ZL and ZDF groups respectively. *: significantly different.

The mitochondrial-derived oxidative stress was estimated in cardiac homogenates by the aconitase-to-fumarase ratio. As shown in Figure 3A, the ratio was significantly reduced in the ZDF group (-41%), indicating an increase in the cardiac mitochondrial oxidative stress. The lactate-to-pyruvate ratio in the coronary effluents (Figure 3B), reflecting the cytosolic redox potential, was also decreased in the ZDF group (-34%). This decrease in the cytosolic redox potential may indicate a diminished capacity of the system to buffer ROS and thus an increased presence of oxidizing species as its primary outcome [30-32]. In the heart, the global antioxidant power or the protein thiol groups were not modified by the diabetic state of the ZDF rats (Figure 4A). However, there was a significant increase of 105% in the lipid peroxidation as shown by the TBARS concentration normalized to the PUFAs content of the cardiac membrane phospholipids (Figure 4B).

In the plasma, even though the antioxidant enzyme GPx was significantly increased in the ZDF group (+21.5%, Figure 3C), the global antioxidant power as estimated by the FRAP assay was significantly decreased (-15,6%, Figure 3D). This was accompanied by a decrease in the plasma thiol groups (Figure 3E), which however did not reach significance.

### Mitochondrial enzymatic activities

Citrate synthase was significantly increased (+4.5%) in the ZDF group as shown by the values for the ZL versus the ZDF rats in Table 2. When normalized to the amount of myocardial proteins, the activity of the cytochrome oxidase was increased in the ZDF group (+20%). No modifications concerning the activities of the other respiratory chain complexes were observed.

**Table 2:** Respiratory chain complex and citrate synthase activities

	**ZL**	**ZDF**
CI	1.06 ± 0.12	1.05 ± 0.08
CII	0.67 ± 0.04	0.68 ± 0.03
CIII	0.22 ± 0.03	0.23 ± 0.02
CIV	0.070 ± 0.006	0.084 ± 0.003*
CI + III	0.040 ± 0.003	0.040 ± 0.003
CII + III	0.022 ± 0.002	0.024 ± 0.002
CS	4.55 ± 0.01	4.75 ± 0.05*

The number of experiments was 11 and 10 for the ZL and ZDF groups respectively. ZL: Zucker lean rats; ZDF: Zucker Diabetic fatty rats; CI: NADH:ubiquinone oxidoreductase; CII: succinate-ubiquinone oxido-reductase; CIII: ubiquinol-cytochrome-c reductase; CIV: cytochrome c oxidase; CI + III: NADH cytochrome c reductase; CII + III: succinate cytochrome c reductase; CS: citrate synthase. The results are expressed in mU/mg of proteins. *: significantly different.

### Fatty acid composition of cardiac phospholipids

The fatty acid composition of cardiac membrane phospholipids was modulated by the development of diabetes (Table 3). The SFAs were significantly increased in the ZDF group (+24%), especially the 18:0 (+33%). This increase partly occurred at the detriment of the MUFAs. Indeed, all the MUFAs were reduced (-36, -27 and -42% for the 16:1n-7, 18:1n-9 and 18:1n-7, respectively). The n-6 PUFAs were also reduced, not only in their totality (-18%) but also regarding the 18:2n-6 (-52%). However, the 20:3 n-6 and 20:4 n-6 were significantly increased (+152 and +47%, respectively). The important reduction of the n-6 PUFAs was accompanied by an increase in n-3 PUFAs (+98%). This was particularly true for the 22:5 n-3 and 22:6 n-3 levels (+130 and + 97%, respectively). Finally, the n-6 to n-3 PUFA ratio of cardiac phospholipids was reduced by the occurrence of diabetes (-60%).

**Table 3:** Fatty acid composition of cardiac phospholipids

**Fatty acids (%)**	**ZL**	**ZDF**
14:0	0.07 ± 0.01	0.05 ± 0.01*
DMA16:0	2.67 ± 0.15	4.39 ± 0.60*
16:0	12.63 ± 0.17	13.25 ± 0.58
DMA18:0	1.20 ± 0.06	1.29 ± 0.25
18:0	19.37 ± 0.50	25.70 ± 1.12*
SFA	35.94 ± 0.80	44.68 ± 1.07*
16:1n-7	0.56 ± 0.02	0.37 ± 0.02*
18:1n-9	3.49 ± 0.16	2.53 ± 0.08*
18:1n-7	5.77 ± 0.22	3.32 ± 0.10*
MUFA	9.81 ± 0.36	6.21 ± 0.16*
18:2n-6	32.94 ± 1.83	15.65 ± 2.57*
20:2n-6	0.17 ± 0.02	0.17 ± 0.01
20:3n-6	0.40 ± 0.05	1.01 ± 0.11*
20:4n-6	16.01 ± 0.83	23.62 ± 1.64*
22:4n-6	0.42 ± 0.04	0.38 ± 0.03
22:5n-6	0.31 ± 0.01	0.39 ± 0.04
n-6 PUFA	50.25 ± 0.94	41.23 ± 1.27*
20:5n-3	0.10 ± 0.01	0.08 ± 0.06
22:5n-3	0.55 ± 0.06	1.26 ± 0.11*
22:6n-3	3.31 ± 0.41	6.51 ± 0.55*
n-3 PUFA	3.95 ± 0.43	7.86 ± 0.64*
PUFA	54.20 ± 0.63	49.09 ± 1.17*
n-6/n-3	13.51 ± 1.70	5.40 ± 0.47*
Total 16:0	15.30 ± 0.28	17.63 ± 0.30*
Total 18:0	20.57 ± 0.54	26.00 ± 0.94*
Total 18:1	9.26 ± 0.35	5.87 ± 0.16*
EPA/AA	0.006 ± 0.001	0.004 ± 0.001*
EPA + DHA	3.4 ± 0.4	6.6 ± 0.6*

Values are expressed as relative amounts of the total fatty acid content. The analysis was performed on 5 samples randomly selected in each group. ZL: Zucker lean rats; ZDF: Zucker diabetic fatty rats; DMA: dimethylacetal; SFA: saturated fatty acids; MUFA: monounsaturated fatty acids; PUFA: polyunsaturated fatty acids; EPA: eicosapentanoic acid; AA: arachidonic acid; DHA: docosahexaenoic acid; *: significantly different.

### Cardiac function study

The results of the *ex vivo* cardiac function are shown in Table 4. The measured parameters were recorded when the heart was perfused at constant flow before the infusion of U46619. In the ZDF group, the RPP was reduced (-35%) compared to the ZL group. This was due to a reduction of the heart rate (-42%), since the left ventricle developed pressure was slightly increased (+18.2%). The changes in the RPP were consequently related to the observed decrease in coronary flow (-25%), but the coronary pressure was unaffected. The infusion of U46619 raised the coronary pressure from 80 mmHg to a value close to 125 mmHg in both groups.

**Table 4:** ***Ex vivo *cardiac function**

	**ZL**	**ZDF**
HR (beats/min)	294 ± 10	171 ± 23*
LVDP (mmHg)	99 ± 6	115 ± 6*
RPP (mHg/min)	29 ± 2	19 ± 2*
CF (ml/min)	14.1 ± 1.0	10.6 ± 0.8*
CP before U46619 (mmHg)	78 ± 8	78 ± 3
CP after U46619 (mmHg)	121 ± 9	130 ± 8

The number of experiments was 11 and 10 for the ZL and ZDF groups respectively. ZL: Zucker lean rats; ZDF: Zucker Diabetic fatty rats; HR: Heart Rate, LVDP: Left Ventricle Developed Pressure, RPP: Rate x Pressure Product, CF: Coronary Flow, CP: Coronary Pressure. * significantly different.

### Coronary reactivity

**Figure 5:**
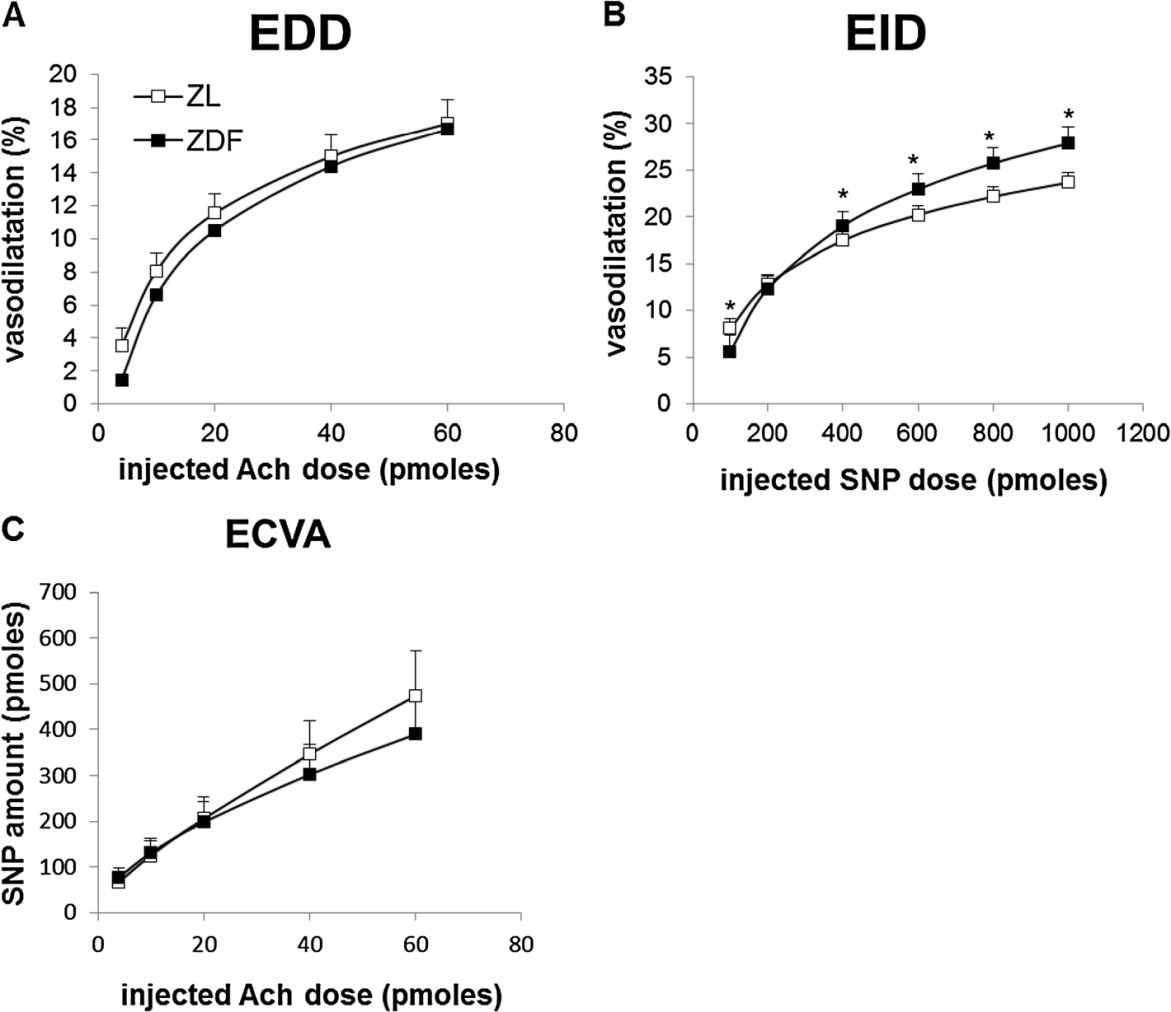
**Coronary microvascular reactivity *ex vivo.*** (**A**) Endothelial-dependent dilatation (EDD). (**B**) Endothelial-independent dilatation (EID). (**C**) Endothelial cell vasodilatation activity (ECVA). ZL: Zucker lean rats; ZDF: Zucker Diabetic Fatty rats; Ach: acetylcholine; SNP: sodium nitroprusside. The number of experiments was 11 and 10 for the ZL and ZDF groups respectively. *: significantly different.

Figure 5A depicts an EDD, which was similar in the two groups, reaching 15% of dilatation as soon as 40 pmoles of Ach were injected. The EID was significantly increased in the ZDF group (Figure 5B) as soon as the SNP dose of 600 pmoles was injected (+14, +16 and +18% at the doses of 600, 800 and 1000 pmoles of SNP, respectively). Finally, the ECVA was not modified by the occurrence of diabetes (Figure 5C).

### eNOS expression and phosphorylation

**Figure 6:**
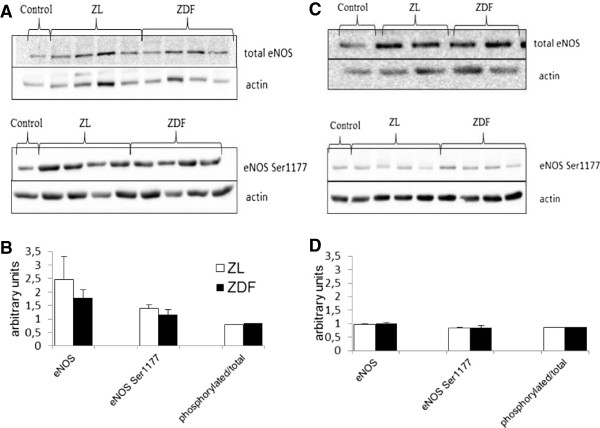
**Protein expressions of total eNOS and phosphorylated eNOS at Ser1177 in aortas and hearts. **(**A**) Representative immunoblots of total eNOS, eNOS phosphorylated at Ser1177 and actin in aorta. Control is the common sample used for all Western blots. (**B**) Quantified total eNOS and phosphorylated eNOS in the aortas and ratio between the phosphorylated and total eNOS in aorta. (**C**) Representative immunoblots of total eNOS, eNOS phosphorylated at Ser1177 and actin in heart. Control is the common sample used for all Western blots. (**D**) Quantified total eNOS and phosphorylated eNOS in the hearts and ratio between the phosphorylated and total eNOS. ZL: Zucker lean rats; ZDF: Zucker Diabetic Fatty rats. The number of experiments was 11 and 10 for the ZL and ZDF groups respectively. *: significantly different.

The eNOS expression and its phosphorylation at Ser1177 were evaluated in aortic and cardiac homogenates of both groups. No difference was observed between groups in the expression and phosphorylation of the enzyme neither in the aorta (Figures 6A and B) nor in the heart (Figures 6C and D).

### Nos2 mRNA and protein expression

**Figure 7:**
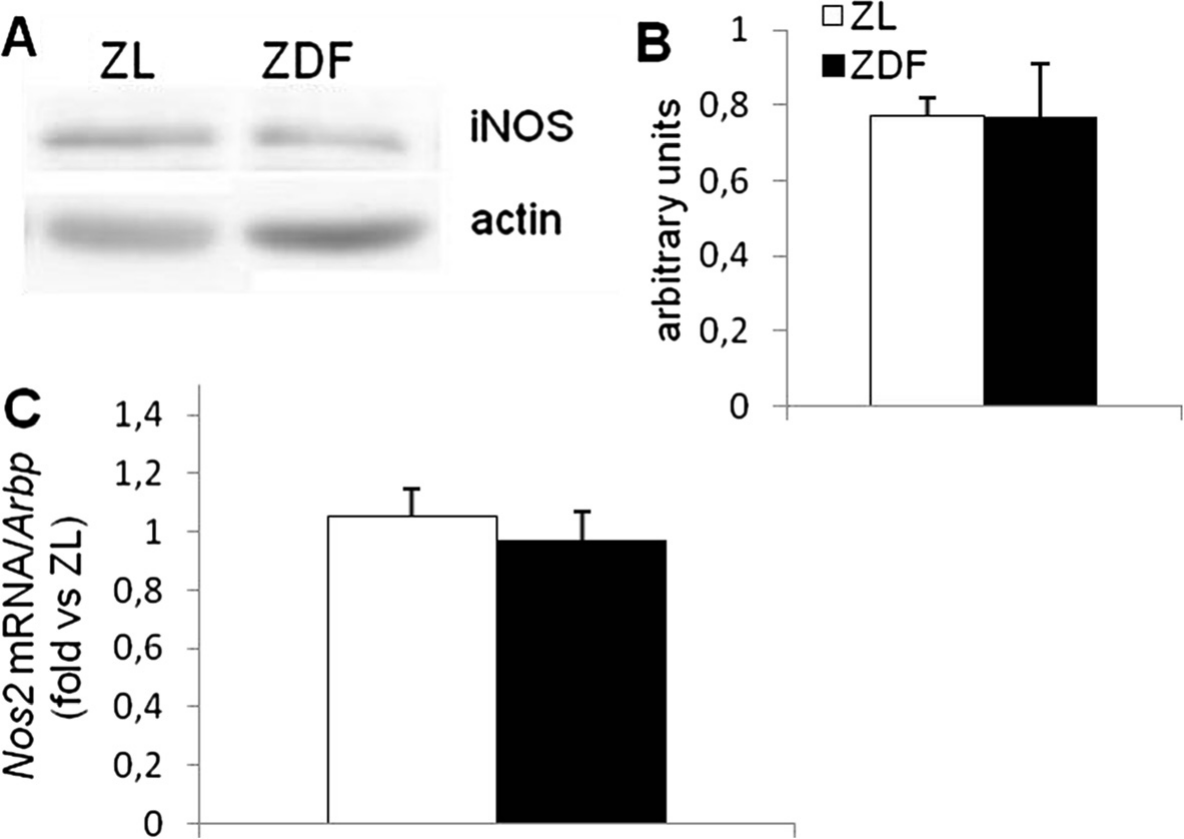
**Expression levels of iNOS protein and Nos2 gene in hearts. **(**A**) Representative immunoblots of iNOS and actin in hearts. (**B**) Quantified iNOS in heart normalized to actin levels. (**C**) mRNA expression levels of *Nos2* expressed in cycle threshold values (Ct) normalized to the house-keeping gene *Arbp*. ZL: Zucker lean rats; ZDF: Zucker Diabetic Fatty rats. The number of experiments was 11 and 10 for the ZL and ZDF groups respectively.

The *Nos2* mRNA and protein expression were evaluated in cardiac homogenates of both groups. No difference was observed between groups at mRNA (Figure 7A) or protein level (Figure 7B).

Surprisingly, after using anti-iNOS antibody, a band of 95-kDa protein instead of 130 kDa was revealed. This could represent a breakdown product of iNOS but the harvest storage and analysis conditions were designed to minimize proteolysis. These experiments were repeated several times but the 130-kDa band never appeared which is consistent with findings by other authors that tried to detect iNOS in cardiac or skeletal muscle tissue [41,42]. It could thus be possible that this 95-kDa band could represent a novel isoform or an alternatively spliced form of iNOS as previously proposed [41]. Furthermore, the evaluation of its mRNA expression indicated the presence of *Nos2* mRNA in the samples and gave results that were in accordance with the Western blot findings.

## Discussion

Even though several studies have examined the effects of diabetes on the vascular function, most of them used techniques of isolated vessels and most of them examined the late stages of diabetes [22,43]. This is the first study that focuses on the effects of T2D on the intact coronary microvasculature at the early phase of the disease. This study addressed cardiac mechanical function in an isolated heart model that provided also the opportunity to study the vascular functionality in the intact coronary circulation. This allowed the analysis of the coupling of cardiac and coronary function, which is not feasible in isolated vessels.

The ZDF rat has been well characterized as experimental model of T2D. The ZL rats in our study ate a normal amount of diet (approximately 20 g/day) and exhibited a low blood glucose concentration (1 g/l) and proportion of HbA1c (approximately 4%) between the 7th and 11th wk of life. Their insulinemia was also low (1 μg/l) at the moment of sacrifice. In contrast, the ZDF animals consumed greater amounts of food (more than 30 g/day) that resulted to a higher body weight during the whole course of the experiment. Their blood analysis revealed a glycemia reaching 5 g/l at the 9th wk of age and a proportion of HbA1c close to 9% representing an already established diabetic state. The insulinemia at the 11th week was almost 4 times higher than that of the ZL control animals, indicating functional β cells in the Langerhans islets despite the high blood glucose concentration. Furthermore, their plasma triglycerides and cholesterol levels were approximately 2 times higher than those of the lean animals. All these characteristics associated with the fact that the visceral fat mass was abnormally high, clearly demonstrate that the ZDF animals displayed a severe insulin resistance responsible for the development of type-2 diabetes, which corresponds to a stage of early human type 2 diabetes.

In our study, the presence of diabetes provoked an enhanced cytosolic and mitochondrial oxidative stress in the hearts of the ZDF rats as observed by the lactate-to-pyruvate and aconitase-to-fumarase ratios respectively. It seems though that the respiratory chain complexes (RCC) were not implicated in the development of this mitochondrial oxidative stress, especially since the complex IV (CIV) activity was increased in the ZDF animals. It has been previously proposed that an up-regulation of CIV activity without any other changes in the other RCC or citrate synthase activities may serve to reduce any production of oxidative stress by the electron transport chain and improve the electron flux [44], but no measurements of mitochondrial respiration were performed in this study in order to directly evaluate the mitochondrial function that could be related to the observed depressed cardiac mechanical work. However, it is probable that a lack of mitochondrial antioxidant defenses could result to the increased mitochondrial oxidative stress observed n this study. The increased oxidative stress was also demonstrated in cardiac level by the TBARS results as already shown in the literature [22]. This could have been the result of the observed increase in the n-3 PUFA content of the cardiac membrane phospholipids of the ZDF hearts, since n-3 PUFAs are highly susceptible to peroxidation. A hyperglycemia-induced ROS production could have resulted to the observed increased lipid peroxidation due to the elevated n-3 PUFA content of membrane phospholipids.

The evaluation of the global plasma antioxidant capacity of ZDF rats was significantly decreased despite the increase of the GPx enzyme activity even though specific activities of other antioxidant enzymes such as thioredoxin reductase or other peroxidases were not determined. This indicates an increased presence of ROS in their plasma as evidenced also by the disappearance of the plasma thiol groups, even though it did not reach absolute significance (p = 0.079, ANOVA). The observed hyperglycemia and hyperinsulinemia could have also induced the NADPH oxidase activity and the consequent production of H_2_O_2_[45] that could explain the increased activity of GPx. The elevated activity of GPx may thus reflect a protective response against increased oxidative stress, since oxidative stress-induced antioxidant adaptive response could be particularly important in high ROS environments. Thus, an increased oxidative stress was already present in the cardiac tissue and plasma at the early phase of T2D despite the effort of the organism (CIV and GPx activities) to eliminate it.

The development of T2D also induced changes in the fatty acid profile of cardiac membrane phospholipids that may influence lipid-protein interactions, inflammation and related metabolic processes. In particular, an increase in the SFAs in cardiac membranes was observed to the detriment of MUFAs. This increased degree of saturation could negatively affect the membrane fluidity and increase its rigidity. However, an increase in the PUFAs content, in particular the C20:4 n-6 (arachidonic acid, AA), C22:5 n-3 (docosapentaenoic acid, DPA) and C22:6 n-3 (docosahexaenoic acid, DHA) contents, was observed probably as an effort to maintain a proper membrane fluidity degree. Moreover, these increases seem to result from stimulation of the desaturation and elongation enzymes in the organism of the ZDF rats rather from an increase in the concentration of the initial phospholipids of the PUFAs metabolisms (e.g. C18:2 n-6 and C20:5 n-3 for n-6 and n-3 respectively). It has been observed that tissues such as heart, kidney and liver from diabetic rats are characterized by a decrease in arachidonylated phospholipids and an increase in phospholipids containing linoleic acid (LA, C18:2 n-6). However, these modifications are mostly related to the later stages of diabetes. The n-3 and n-6 PUFAs of membrane phospholipids are also responsible for the production of anti- and pro-inflammatory molecules respectively. The low ratio EPA (eicosapentanoic acid)/AA found in our ZDF rats predisposes to a balance of eicosanoids favouring platelet aggregation and inflammatory mediator signaling [46]. The development of a compensatory mechanism might thus be in question as the levels of n-3 PUFAs were increased in the ZDF group. Low levels of EPA + DHA have been related to increased risk for sudden cardiac death [46] and hearts with high DHA content present very low *in vivo* and *in vitro* vulnerability to arrhythmia [47]. The ZDF hearts have high levels of EPA + DHA in order to reduce pro-inflammatory eicosanoids and cytokines. These modifications in the PUFAs levels of the cardiac membrane phospholipids probably help the heart to resist to any sudden cardiac damage at this early phase of diabetes [47].

In our study, we reported a strong decrease in the *ex vivo* cardiac function as already shown in pre-diabetic [48] and diabetic [49,50] states, even though Daniels *et al.*[51] did not found an impaired *in vivo* cardiac function in db/db mice until 18 weeks of age. This could reflect the fact that in our study we have used an *ex vivo* heart perfusion model where the perfusion buffer is similar for both control and diabetic groups while the diabetic hearts *in vivo* are submitted to different plasma substrate and hormone concentrations than the control ones. In our *ex vivo* model, the RPP was significantly reduced, mainly because of the decreased heart rate. The T2D-induced reduction of the heart rate has already been commonly shown in the diabetic state [52] and it has been explained by an abnormal functioning of the cells involved in the generation and transfer of the electric influx triggering the cardiac contraction [53]. We also observed a noticeable diabetes-induced decrease in the coronary flow. This decrease could also be responsible for the reduction of the heart rate and cardiac mechanical work through insufficient oxygen supply. This could not be explained by an increase in the vascular tone triggering vasoconstriction and limitation of the oxygen and substrate supply since no abnormalities of the vascular function were observed according to the results of the vascular reactivity. Finally, the two phenomena could be synergistic and lead to the decreased RPP. In contrast, the left ventricle developed pressure was increased, which could compensate for the decreased heart rate. This phenomenon could be related to the action of sarcoplasmic reticulum Ca^2+^ ATPase (SERCA) whose levels have been shown to be increased in early diabetes and especially stimulated by the presence of insulin [54] but were not evaluated in this work. Thus, our data confirm a decrease in the *ex vivo* cardiac function, and particularly in the heart rate, at this model of heart perfusion, which is a common characteristic of all types of diabetes.

The underlying mechanism, which could explain the observed decreased cardiac mechanical work, has been already characterized. Several metabolic modifications in our study suggest that the reduced *ex vivo* cardiac function was due to this mechanism. In this study, we found an increased plasma triglyceride concentration that could allow the excess free fatty acid uptake and stimulation of the peroxisome proliferator-activated receptor alpha (PPARα) [50]. This would lead to increased β-oxidation and mitochondrial oxygen consumption [55]. The resulting excessive mitochondria-related ROS production, as evidenced by the aconitase-to-fumarase ratio in our study, would favour the expression of protein 53 (p53). The observed increased activity of the cytochrome c oxidase suggests an increased expression of the cytochrome c oxidase 2 (SCO2). Consequently, ectopic lipid accumulation may occur in the cardiomyocytes through increased expression of the fatty acid translocase protein FAT/CD36. Lipotoxicity then contributes to cardiac cell damages and myocardial dysfunction. A severe intramyocardial lipid accumulation, even at 8 wk of age [56] and an increased fatty acid oxidation [49] have been observed in ZDF rats. Thus, the altered myocardial substrate utilization affecting the mitochondrial function and stimulating the above described mechanism could be one of the factors responsible for the development of the T2D-induced *ex vivo* cardiac mechanical dysfunction leading to reduction of oxygen demand and subsequent decrease in the coronary perfusion. In our study though we did not observe any decrease in the left ventricle developed pressure that could have resulted from this mechanism. Instead, the heart rate was the parameter mostly affected by the diabetes in our study. However, the conditions of the perfusion model did not allow us to evaluate correctly the left ventricle developed pressure. A future study of heart perfusion at stable heart rate (pacing) in order to evaluate the cardiac contractility and relaxation by measuring the maximal rate of the ventricular pressure rise (dP/dt_max_) and fall (dP/dt_min_) respectively could enlighten our knowledge concerning this mechanism. Taken together, these observations suggest that the lipid accumulation and alterations of substrate utilization in ZDF rats may affect firstly the cells responsible for the cardiac contraction that are involved in the generation and transfer of the electric influx. Furthermore, hyperglycemia and insulin resistance, two states that characterized the ZDF rats in this study, have been related to damages in cardiac nodal cells and to cardiac electrophysiological properties.

The effects of the T2D on the function of the coronary resistance arteries were also evaluated in this *ex vivo* model of isolated perfused heart through measurement of the coronary reactivity. This parameter was estimated through changes in the global coronary tone which mainly reflects the function of the arteriole network, since atherosclerosis does not occur in the rat [57]. The effects of T2D on the heart can thus be evaluated independently of the development of coronary artery disease. In this study, the EDD of diabetic hearts was fully maintained while the EID was even enhanced. We also evaluated indirectly the NO production through measurements of the expression and phosphorylation of the cardiac and aortic eNOS, since the phosphorylation of the enzyme at this site has been shown to increase NO production [58], and that of the iNOS levels, since it has been shown that under diabetic conditions stress-induced iNOS is able to produce an abnormal amount of NO [59]. In our study though, diabetes did not modify neither cardiac iNOS levels nor the expression and phosphorylation of eNOS at Ser1177, even though studies concerning other phosphorylation sites or the cGMP signaling pathway were not performed. It has been recently shown that the formation of superoxide from uncoupled bovine eNOS in endothelial cell can be stimulated by this kind of phosphorylation [60]. The presence of increased oxidative stress in the diabetic hearts of the ZDF rats could have led to the depletion of tetrahydrobiopterin (BH_4_) and uncoupling of the eNOS. This could result to increased production of ROS from the enzyme even though there was no change in the phosphorylation of eNOS at Ser1177. The consequent superoxide production could lead to H_2_O_2_ production, which could have participated to the maintained EDD of our study since it has been shown that this molecule can act as endothelium-hyperpolarizing factor (EDHF) [61]. The maintained EDD was a surprising finding given the huge amount of studies associating T2D and dysfunctions of the coronary microcirculation [62-64]. Factors contributing to these discrepancies are the severity of the obesity and diabetic state studied as well as the experimental method used. Oltman *et al.*[22] have reported a preservation of the coronary arteriolar dilatation to Ach in isolated vessels of pre-diabetic young (8- to 12-wk old) ZDF rats. However, in the present study, the ZDF rats were not in a prediabetic state, but the T2D was already developed as indicated by the blood glucose concentration, which was already high from the 8th wk of age. Thus, it seems that the endothelial function of the intact coronary microvasculature is not affected from the diabetes at this phase. The unaltered eNOS and iNOS activities and the high levels of AA and DHA despite the presence of oxidative stress found in the diabetic hearts could have contributed to this phenomenon. AA [65] and DHA [66] are known for their vasorelaxant effects via the production of prostacyclin (PGI2) and the reduction of calcium influx in vascular smooth muscle cells. A future study of vascular reactivity in the presence of a cyclo-oxygenase (COX) inhibitor such as indomethacin could enlighten this hypothesis. Furthermore, the early diabetes could have provoked an increased expression of SERCA [54] favouring the calcium uptake in vascular smooth muscle cells that could be involved in the increased EID observed in the ZDF diabetic hearts of this study.

As shown by the calculated activity of endothelial cells to induce dilatation and the evaluation of eNOS expression the phenomenon of the maintained Ach-mediated vasodilatation was partly mediated by the activity of endothelial cells. However, the SNP responses were enhanced in the ZDF rats representing an enhanced function of the smooth muscle cells of the coronary system contributing to the maintained endothelium-dependent dilatation. This enhanced function may be due to a modified NO response, which could increase guanylate cyclase activity as already shown in cases of obesity and hypertension [67,68].

These vascular alterations may reflect a compensatory adaptation of the cardiovascular system to support increased cardiac work since cardiac output and stroke volume are increased in obese and diabetic states [69,70]. Taken also under consideration the decreased *ex vivo* cardiac mechanical function observed in this study, this adaptation seems to be essential to adjust organ perfusion during physiological processes such as exercise and pathological processes such as ischemic diseases [11]. Otherwise, the heart would not be able to respond to the increased metabolic demands. These results come in agreement with data of Oltman *et. al*[22] that showed that there is no change in the Ach response of small coronary arteries in ZDF rats at the age of 8–12 weeks but there is a progressive impairment until the age of 40 weeks.

## Conclusions

Cardiovascular function was evaluated in young diabetic ZDF rats using an *ex vivo* heart perfusion model. Our data suggest that at the early phase of diabetes, increased oxidative stress in tissue and plasma is already present and probably responsible for the observed *ex vivo* cardiac mechanical dysfunction. However, the heart tries to resist by preserving the EDD of the coronary microvasculature. A number of other adaptations seem to take place at this phase of the disease such as the increased GPx and CIV activities and the increase in the n-3 PUFAs content of the myocardial membrane. This would help the heart to keep an adequate perfusion and respond to any acute cardiac incident at this phase. Thus, therapeutic interventions at this early phase of the disease aiming at increasing the heart rate and maintaining the observed adaptations could be an option for delaying or decreasing the late-stage complications of the diabetes.

## Abbreviations

Arbp: Acidic ribosomal protein; AA: Arachidonic acid; Ach: Acetylcholine; BH4: Tetrahydrobiopterin; CIV: Complex IV; cGMP: Cyclic guanosin monophosphate; COX: Cyclo-oxygenase; CVDs: Cardiovascular diseases; DHA: Docosahexanoic acid; DPA: Docosapentaenoic acid; EDHF: Endothelium-derived hyperpolarizing factor; EPA: Eicosapentanoic acid; ECVA: Endothelial cell vasodilatation activity; EDD: Endothelial-dependent vasodilatation; EID: Endothelial-independent vasodilatation; eNOS: Endothelial nitric oxide synthase; FAT/CD36: Fatty acid translocase protein; FRAP: Ferric reducing antioxidant power assay; GPx: Glutathione peroxidase; GR: Glutathione reductase; GSH: Glutathione; GSSG: Oxidized glutathione; H2O2: Hydrogen peroxide; HbA1c: Glycated hemoglobin; iNOS/Nos2: Nitric oxide synthase 2 inducible; LA: Linoleic acid; LDH: Lactate dehydrogenase; MUFAs: Monounsaturated fatty acids; NO: Nitric Oxide; PGI2: Prostacyclin; PPARα: Peroxisome proliferator receptor alpha; PUFAs: Polyunsaturated fatty acids; RCC: Respiratory chain complexes; ROS: Reactive oxygen species; RPP: Rate-pressure product; SERCA: Sarcoplasmic reticulum Ca^2+^ ATPase; SCO2: Cytochrome C oxidase 2; Ser1177: Serine 1177; SFAs: Saturated fatty acids; SNP: Sodium nitroprusside; TBARS: Thiobarbituric reactive substances; T2D: Type 2 diabetes; ZDF: Zucker diabetic fatty rats; ZL: Zucker lean rats.

## Competing interests

The authors declare that they have no competing interests.

## Authors’ contributions

Evangelia Mourmoura conducted the experiments and contributed to the study implementation, statistical analysis, interpretation, and the preparation of the manuscript. Guillaume Vial conducted a part of biochemical experiments and participated in the animal care. Brigitte Laillet, Jean-Paul Rigaudière, Isabelle Hininger, Hervé Dubouchaud and Beatrice Morio helped to conduct the experiments and acquire data. Luc Demaison supervised the study conduction and contributed to the study conception and design, implementation, statistical interpretation, the preparation and finalization of the manuscript. All authors approved the final manuscript for publication.

## References

[B1] Wild S Roglic G Green A Sicree R King H Global prevalence of diabetes: estimates for the year 2000 and projections for 2030 Diabetes Care 2004 27 5 1047 1053 10.2337/diacare.27.5.1047 15111519

[B2] Mathers CD Loncar D Projections of global mortality and burden of disease from 2002 to 2030 PLoS medicine 2006 3 11 e442 10.1371/journal.pmed.0030442 17132052 PMC1664601

[B3] Funaki M Saturated fatty acids and insulin resistance J Med Invest 2009 56 3–4 88 92 19763019 10.2152/jmi.56.88

[B4] Goldstein BJ Insulin resistance as the core defect in type 2 diabetes mellitus Am J Cardiol 2002 90 5A 3G 10G 12231073 10.1016/s0002-9149(02)02553-5

[B5] Sowers JR Epstein M Frohlich ED Diabetes, hypertension, and cardiovascular disease: an update Hypertension 2001 37 4 1053 1059 10.1161/01.HYP.37.4.1053 11304502

[B6] Chinen I Shimabukuro M Yamakawa K Higa N Matsuzaki T Noguchi K Ueda S Sakanashi M Takasu N Vascular lipotoxicity: endothelial dysfunction via fatty-acid-induced reactive oxygen species overproduction in obese zucker diabetic fatty rats Endocrinology 2007 148 1 160 165 17023526 10.1210/en.2006-1132

[B7] Heitzer T Schlinzig T Krohn K Meinertz T Munzel T Endothelial dysfunction, oxidative stress, and risk of cardiovascular events in patients with coronary artery disease Circulation 2001 104 22 2673 2678 10.1161/hc4601.099485 11723017

[B8] Thuillez C Richard V Targeting endothelial dysfunction in hypertensive subjects J Hum Hypertens 2005 19 Suppl 1 S21 S25 16075029 10.1038/sj.jhh.1001889

[B9] Nitenberg A Valensi P Sachs R Dali M Aptecar E Attali JR Impairment of coronary vascular reserve and ACh-induced coronary vasodilation in diabetic patients with angiographically normal coronary arteries and normal left ventricular systolic function Diabetes 1993 42 7 1017 1025 10.2337/diabetes.42.7.1017 8513969

[B10] Clark JB Palmer CJ Shaw WN The diabetic zucker fatty rat Proceedings of the Society for Experimental Biology and Medicine Society for Experimental Biology and Medicine 1983 173 1 68 75 10.3181/00379727-173-41611 6344096

[B11] Wang P Chatham JC Onset of diabetes in zucker diabetic fatty (ZDF) rats leads to improved recovery of function after ischemia in the isolated perfused heart Am J Physiol Endocrinol Metab 2004 286 5 E725 E736 10.1152/ajpendo.00295.2003 14722022

[B12] Greenberg AS McDaniel ML Identifying the links between obesity, insulin resistance and beta-cell function: potential role of adipocyte-derived cytokines in the pathogenesis of type 2 diabetes Eur J Clin Investig 2002 32 Suppl 3 24 34 12028372 10.1046/j.1365-2362.32.s3.4.x

[B13] Bastard JP Maachi M Van Nhieu JT Jardel C Bruckert E Grimaldi A Robert JJ Capeau J Hainque B Adipose tissue IL-6 content correlates with resistance to insulin activation of glucose uptake both *in vivo* and *in vitro* J Clin Endocrinol Metab 2002 87 5 2084 2089 10.1210/jc.87.5.2084 11994345

[B14] Erdos B Snipes JA Miller AW Busija DW Cerebrovascular dysfunction in zucker obese rats is mediated by oxidative stress and protein kinase C Diabetes 2004 53 5 1352 1359 10.2337/diabetes.53.5.1352 15111506

[B15] Oltman CL Coppey LJ Gellett JS Davidson EP Lund DD Yorek MA Progression of vascular and neural dysfunction in sciatic nerves of zucker diabetic fatty and zucker rats Am J Physiol Endocrinol Metab 2005 289 1 E113 E122 10.1152/ajpendo.00594.2004 15727946

[B16] Coppey LJ Gellett JS Davidson EP Dunlap JA Yorek MA Changes in endoneurial blood flow, motor nerve conduction velocity and vascular relaxation of epineurial arterioles of the sciatic nerve in ZDF-obese diabetic rats Diabetes Metab Res Rev 2002 18 1 49 56 10.1002/dmrr.257 11921418

[B17] Cai H Harrison DG Endothelial dysfunction in cardiovascular diseases: the role of oxidant stress Circ Res 2000 87 10 840 844 10.1161/01.RES.87.10.840 11073878

[B18] Pieper GM Langenstroer P Siebeneich W Diabetic-induced endothelial dysfunction in rat aorta: role of hydroxyl radicals Cardiovasc Res 1997 34 1 145 156 10.1016/S0008-6363(96)00237-4 9217884

[B19] Winer N Sowers JR Diabetes and arterial stiffening Adv Cardiol 2007 44 245 251 17075213 10.1159/000096745

[B20] Hattori Y Kawasaki H Abe K Kanno M Superoxide dismutase recovers altered endothelium-dependent relaxation in diabetic rat aorta Am J Physiol 1991 261 4 Pt 2 H1086 H1094 1928390 10.1152/ajpheart.1991.261.4.H1086

[B21] Chilian WM Coronary microcirculation in health and disease. Summary of an NHLBI workshop Circulation 1997 95 2 522 528 10.1161/01.CIR.95.2.522 9008472 PMC4037233

[B22] Oltman CL Richou LL Davidson EP Coppey LJ Lund DD Yorek MA Progression of coronary and mesenteric vascular dysfunction in zucker obese and zucker diabetic fatty rats Am J Physiol Heart Circ Physiol 2006 291 4 H1780 H1787 10.1152/ajpheart.01297.2005 16714356

[B23] Kilkenny C Browne WJ Cuthill IC Emerson M Altman DG Improving bioscience research reporting: the ARRIVE guidelines for reporting animal research PLoS biology 2010 8 6 e1000412 10.1371/journal.pbio.1000412 20613859 PMC2893951

[B24] Fu WJ Haynes TE Kohli R Hu J Shi W Spencer TE Carroll RJ Meininger CJ Wu G Dietary L-arginine supplementation reduces fat mass in zucker diabetic fatty rats J Nutr 2005 135 4 714 721 15795423 10.1093/jn/135.4.714

[B25] Skrzypiec-Spring M Grotthus B Szelag A Schulz R Isolated heart perfusion according to langendorff–-still viable in the new millennium Journal of pharmacological and toxicological methods 2007 55 2 113 126 10.1016/j.vascn.2006.05.006 16844390

[B26] Gobel FL Norstrom LA Nelson RR Jorgensen CR Wang Y The rate-pressure product as an index of myocardial oxygen consumption during exercise in patients with angina pectoris Circulation 1978 57 3 549 556 10.1161/01.CIR.57.3.549 624164

[B27] Faure P Lafond J Favier A, Cadet J, Kalnyanaraman M, Fontecave M, Pierre J Measurement of plasma sulfhydryl and carbonyl groups as a possible indicator of protein oxidation Analysis of free radicals in biological systems 1995 Basel: Birkhauser 237 248

[B28] Gunzler WA Kremers H Flohe L An improved coupled test procedure for glutathione peroxidase (EC 1-11-1-9-) in blood Z Klin Chem Klin Biochem 1974 12 10 444 448 4154542 10.1515/cclm.1974.12.10.444

[B29] Bergmeyer HU Gawehn K Williamson DH Lund P Methods of enzymatic analysis 1974 2 English Weinheim New York; London: Verlag Chemie: Academic

[B30] Nuutinen EM Subcellular origin of the surface fluorescence of reduced nicotinamide nucleotides in the isolated perfused rat heart Basic Res Cardiol 1984 79 1 49 58 10.1007/BF01935806 6233965

[B31] Goodwin GW Taylor CS Taegtmeyer H Regulation of energy metabolism of the heart during acute increase in heart work J Biol Chem 1998 273 45 29530 29539 10.1074/jbc.273.45.29530 9792661

[B32] Baumberger JP Jurgensen JJ Bardwell K The coupled redox potential of the lactate-enzyme-pyruvate system J Gen Physiol 1933 16 6 961 976 10.1085/jgp.16.6.961 19872753 PMC2141256

[B33] Richard MJ Portal B Meo J Coudray C Hadjian A Favier A Malondialdehyde kit evaluated for determining plasma and lipoprotein fractions that react with thiobarbituric acid Clin Chem 1992 38 5 704 709 1582024

[B34] Gardner PR Nguyen DD White CW Aconitase is a sensitive and critical target of oxygen poisoning in cultured mammalian cells and in rat lungs Proc Natl Acad Sci U S A 1994 91 25 12248 12252 10.1073/pnas.91.25.12248 7991614 PMC45414

[B35] Mourmoura E Leguen M Dubouchaud H Couturier K Vitiello D Lafond JL Richardson M Leverve X Demaison L Middle age aggravates myocardial ischemia through surprising upholding of complex II activity, oxidative stress, and reduced coronary perfusion Age 2011 33 3 321 336 10.1007/s11357-010-9186-0 20878490 PMC3168590

[B36] Faloona GR Srere PA Escherichia coli citrate synthase. Purification and the effect of potassium on some properties Biochemistry 1969 8 11 4497 4503 10.1021/bi00839a041 4900996

[B37] Livak KJ Schmittgen TD Analysis of relative gene expression data using real-time quantitative PCR and the 2(-delta delta C(T)) method Methods 2001 25 4 402 408 10.1006/meth.2001.1262 11846609

[B38] Demaison L Moreau D Vergely-Vandriesse C Gregoire S Degois M Rochette L Effects of dietary polyunsaturated fatty acids and hepatic steatosis on the functioning of isolated working rat heart under normoxic conditions and during post-ischemic reperfusion Mol Cell Biochem 2001 224 1–2 103 116 11693187 10.1023/a:1011934603667

[B39] Folch J Lees M Sloane Stanley GH A simple method for the isolation and purification of total lipides from animal tissues J Biol Chem 1957 226 1 497 509 13428781

[B40] Juaneda P Rocquelin G Rapid and convenient separation of phospholipids and non phosphorus lipids from rat heart using silica cartridges Lipids 1985 20 1 40 41 10.1007/BF02534360 2982073

[B41] Sudar E Dobutovic B Soskic S Mandusic V Zakula Z Misirkic M Vucicevic L Janjetovic K Trajkovic V Mikhailidis DP Regulation of inducible nitric oxide synthase activity/expression in rat hearts from ghrelin-treated rats J Physiol Biochem 2011 67 2 195 204 10.1007/s13105-010-0063-1 21107779

[B42] Wang MX Murrell DF Szabo C Warren RF Sarris M Murrell GA Nitric oxide in skeletal muscle: inhibition of nitric oxide synthase inhibits walking speed in rats Nitric oxide: biology and chemistry/official journal of the Nitric Oxide Society 2001 5 3 219 232 10.1006/niox.2001.0348 11384195

[B43] Okon EB Szado T Laher I McManus B van Breemen C Augmented contractile response of vascular smooth muscle in a diabetic mouse model J Vasc Res 2003 40 6 520 530 10.1159/000075238 14646372

[B44] Parise G Brose AN Tarnopolsky MA Resistance exercise training decreases oxidative damage to DNA and increases cytochrome oxidase activity in older adults Exp Gerontol 2005 40 3 173 180 10.1016/j.exger.2004.09.002 15763394

[B45] Goldstein BJ Mahadev K Wu X Zhu L Motoshima H Role of insulin-induced reactive oxygen species in the insulin signaling pathway Antioxid Redox Signal 2005 7 7–8 1021 1031 15998257 10.1089/ars.2005.7.1021 PMC1434604

[B46] Rupp H Wagner D Rupp T Schulte LM Maisch B Risk stratification by the "EPA + DHA level" and the "EPA/AA ratio" focus on anti-inflammatory and antiarrhythmogenic effects of long-chain omega-3 fatty acids Herz 2004 29 7 673 685 10.1007/s00059-004-2602-4 15580322

[B47] Pepe S McLennan PL Cardiac membrane fatty acid composition modulates myocardial oxygen consumption and postischemic recovery of contractile function Circulation 2002 105 19 2303 2308 10.1161/01.CIR.0000015604.88808.74 12010914

[B48] Essop MF Anna Chan WY Valle A Garcia-Palmer FJ Du Toit EF Impaired contractile function and mitochondrial respiratory capacity in response to oxygen deprivation in a rat model of pre-diabetes Acta Physiol (Oxf) 2009 197 4 289 296 10.1111/j.1748-1716.2009.02024.x 19645752

[B49] Wang P Lloyd SG Zeng H Bonen A Chatham JC Impact of altered substrate utilization on cardiac function in isolated hearts from zucker diabetic fatty rats Am J Physiol Heart Circ Physiol 2005 288 5 H2102 H2110 10.1152/ajpheart.00935.2004 15615844

[B50] Finck BN Han X Courtois M Aimond F Nerbonne JM Kovacs A Gross RW Kelly DP A critical role for PPARalpha-mediated lipotoxicity in the pathogenesis of diabetic cardiomyopathy: modulation by dietary fat content Proc Natl Acad Sci U S A 2003 100 3 1226 1231 10.1073/pnas.0336724100 12552126 PMC298755

[B51] Daniels A van Bilsen M Janssen BJ Brouns AE Cleutjens JP Roemen TH Schaart G van der Velden J van der Vusse GJ van Nieuwenhoven FA Impaired cardiac functional reserve in type 2 diabetic db/db mice is associated with metabolic, but not structural, remodelling Acta Physiol (Oxf) 2010 200 1 11 22 20175764 10.1111/j.1748-1716.2010.02102.x

[B52] Semeniuk LM Kryski AJ Severson DL Echocardiographic assessment of cardiac function in diabetic db/db and transgenic db/db-hGLUT4 mice Am J Physiol Heart Circ Physiol 2002 283 3 H976 H982 12181126 10.1152/ajpheart.00088.2002

[B53] Barth AS Tomaselli GF Cardiac metabolism and arrhythmias Circulation Arrhythmia and electrophysiology 2009 2 3 327 335 10.1161/CIRCEP.108.817320 19808483 PMC2744981

[B54] Fredersdorf S Thumann C Zimmermann WH Vetter R Graf T Luchner A Riegger GA Schunkert H Eschenhagen T Weil J Increased myocardial SERCA expression in early type 2 diabetes mellitus is insulin dependent: *In vivo* and *in vitro* data Cardiovasc Diabetol 2012 11 57 10.1186/1475-2840-11-57 22621761 PMC3447673

[B55] Nakamura H Matoba S Iwai-Kanai E Kimata M Hoshino A Nakaoka M Katamura M Okawa Y Ariyoshi M Mita Y p53 Promotes cardiac dysfunction in diabetic mellitus caused by excessive mitochondrial respiration-mediated reactive oxygen species generation and lipid accumulation Circ Heart Fail 2012 5 1 106 115 10.1161/CIRCHEARTFAILURE.111.961565 22075967

[B56] Sharma S Adrogue JV Golfman L Uray I Lemm J Youker K Noon GP Frazier OH Taegtmeyer H Intramyocardial lipid accumulation in the failing human heart resembles the lipotoxic rat heart FASEB J 2004 18 14 1692 1700 10.1096/fj.04-2263com 15522914

[B57] Wissler RW The production of atheromatous lesions in the albino rat Proc Inst Med Chic 1952 19 4 79 80 14920402

[B58] McCabe TJ Fulton D Roman LJ Sessa WC Enhanced electron flux and reduced calmodulin dissociation may explain "calcium-independent" eNOS activation by phosphorylation J Biol Chem 2000 275 9 6123 6128 10.1074/jbc.275.9.6123 10692402

[B59] Nagareddy PR Xia Z McNeill JH MacLeod KM Increased expression of iNOS is associated with endothelial dysfunction and impaired pressor responsiveness in streptozotocin-induced diabetes Am J Physiol Heart Circ Physiol 2005 289 5 H2144 H2152 10.1152/ajpheart.00591.2005 16006542

[B60] Chen CA Druhan LJ Varadharaj S Chen YR Zweier JL Phosphorylation of endothelial nitric-oxide synthase regulates superoxide generation from the enzyme J Biol Chem 2008 283 40 27038 27047 10.1074/jbc.M802269200 18622039 PMC2556006

[B61] Shimokawa H Matoba T Hydrogen peroxide as an endothelium-derived hyperpolarizing factor Pharmacol Res 2004 49 6 543 549 10.1016/j.phrs.2003.10.016 15026032

[B62] Gao X Picchi A Zhang C Upregulation of TNF-alpha and receptors contribute to endothelial dysfunction in zucker diabetic rats Am J Biomed Sci 2010 2 1 1 12 20559450 10.5099/aj100100001 PMC2886289

[B63] Oltman CL Kleinschmidt TL Davidson EP Coppey LJ Lund DD Yorek MA Treatment of cardiovascular dysfunction associated with the metabolic syndrome and type 2 diabetes Vascul Pharmacol 2008 48 1 47 53 10.1016/j.vph.2007.11.005 18164667

[B64] Oniki H Fujii K Kansui Y Goto K Iida M Effects of angiotensin II receptor antagonist on impaired endothelium-dependent and endothelium-independent relaxations in type II diabetic rats J Hypertens 2006 24 2 331 338 10.1097/01.hjh.0000200518.34980.cc 16508581

[B65] Spector AA Hoak JC Fry GL Denning GM Stoll LL Smith JB Effect of fatty acid modification on prostacyclin production by cultured human endothelial cells J Clin Invest 1980 65 5 1003 1012 10.1172/JCI109752 6767738 PMC371430

[B66] Mori TA Watts GF Burke V Hilme E Puddey IB Beilin LJ Differential effects of eicosapentaenoic acid and docosahexaenoic acid on vascular reactivity of the forearm microcirculation in hyperlipidemic, overweight men Circulation 2000 102 11 1264 1269 10.1161/01.CIR.102.11.1264 10982541

[B67] Brandes RP Kim D Schmitz-Winnenthal FH Amidi M Godecke A Mulsch A Busse R Increased nitrovasodilator sensitivity in endothelial nitric oxide synthase knockout mice: role of soluble guanylyl cyclase Hypertension 2000 35 1 Pt 2 231 236 10642303 10.1161/01.hyp.35.1.231

[B68] Jebelovszki E Kiraly C Erdei N Feher A Pasztor ET Rutkai I Forster T Edes I Koller A Bagi Z High-fat diet-induced obesity leads to increased NO sensitivity of rat coronary arterioles: role of soluble guanylate cyclase activation Am J Physiol Heart Circ Physiol 2008 294 6 H2558 H2564 10.1152/ajpheart.01198.2007 18408126

[B69] Radovits T Korkmaz S Loganathan S Barnucz E Bomicke T Arif R Karck M Szabo G Comparative investigation of the left ventricular pressure-volume relationship in rat models of type 1 and type 2 diabetes mellitus Am J Physiol Heart Circ Physiol 2009 297 1 H125 H133 10.1152/ajpheart.00165.2009 19429826

[B70] Crandall DL Goldstein BM Lizzo FH Gabel RA Cervoni P Hemodynamics of obesity: influence of pattern of adipose tissue cellularity Am J Physiol 1986 251 2 Pt 2 R314 R319 3740313 10.1152/ajpregu.1986.251.2.R314

